# MAD2L2 inhibits colorectal cancer growth by promoting NCOA3 ubiquitination and degradation

**DOI:** 10.1002/1878-0261.12173

**Published:** 2018-02-13

**Authors:** Yixin Li, Liren Li, Miao Chen, Xinfa Yu, Zhuoyu Gu, Huijuan Qiu, Ge Qin, Qian Long, Xiaoyan Fu, Tianze Liu, Wenbin Li, Wenlin Huang, Dingbo Shi, Tiebang Kang, Meihua Luo, Xiaojun Wu, Wuguo Deng

**Affiliations:** ^1^ Sun Yat‐sen University Cancer Center State Key Laboratory of Oncology in South China Collaborative Innovation Center of Cancer Medicine Guangzhou China; ^2^ Shunde Hospital of Southern Medical University Foshan China; ^3^ Department of Pharmacology Medical College Jinan University Guangzhou China; ^4^ State Key Laboratory of Targeted Drug for Tumors of Guangdong Province Guangzhou Double Bioproduct Inc. Guangzhou China

**Keywords:** colorectal cancer, degradation, MAD2L2, NCOA3, ubiquitination

## Abstract

Nuclear receptor coactivator 3 (NCOA3) is a transcriptional coactivator that has elevated expression in multiple tumor types, including colorectal cancer (CRC). However, the molecular mechanisms that regulate the tumorigenic functions of NCOA3 in CRC remain largely unknown. In this study, we aimed to discover and identify the novel regulatory proteins of NCOA3 and explore their mechanisms of action. Immunoprecipitation (IP) coupled with mass spectrometry (IP‐MS) analysis was used to detect, identify, and verify the proteins that interacted with NCOA3 in CRC cells. The biological functions of the candidate proteins and the underlying molecular mechanism were investigated in CRC cells and mouse model *in vitro* and *in vivo*. The clinical significance of NCOA3 and its interaction partner protein in CRC patients was also studied. We identified mitotic arrest deficient 2‐like protein 2 (MAD2L2, also known as MAD2B or REV7), with two signal peptide sequences of LIPLK and EVYPVGIFQK, to be an interaction partner of NCOA3. Overexpression of MAD2L2 suppressed the proliferation, migration, and clonogenicity of CRC cells by inducing the degradation of NCOA3. The mechanism study showed that increased MAD2L2 expression in CRC cells activated p38, which was required for the phosphorylation of NCOA3 that led to its ubiquitination and degradation by the proteasome. Moreover, we found that MAD2L2 predicted favorable prognosis in CRC patients. We have discovered a novel role of MAD2L2 in the regulation of NCOA3 degradation and proposed that MAD2L2 serves as a tumor suppressor in CRC.

**Abbreviations**
ANT: adjacent normal tissues
CHX: cycloheximide
CIN: chromosomal instability
CRC: colorectal cancer
ERK: Extracellular signal‐regulated kinase
ESCs: embryonic stem cells
HAT: histone acetyltransferase
HR: homologous recombination
IHC: Immunohistochemical
JNK: c‐Jun N‐terminal protein kinase
LOH: loss of heterozygosity
MAD2L2: mitotic arrest deficient 2‐like protein 2
MMS: Methyl methanesulfonate
MS: mass spectrometry
MSI: microsatellite instability
NCOA3: Nuclear receptor coactivator 3
NHEJ: nonhomologous end joining
TLS: translesion synthesis

## 1 Introduction

Colorectal cancer (CRC) is the second most common cancer in women and the third most common in men (Tariq and Ghias, 2016), with global incidence, mortality, and 5‐year prevalence of 9.7%, 8.5%, and 10.9%, respectively, according to GLOBOCAN 2012 (Ferlay *et al*., 2015). The molecular mechanisms in colorectal cancer development include chromosomal instability (CIN) (Grady and Carethers, 2008), microsatellite instability (MSI) (Walther *et al*., 2009), and CpG island methylation (CIMP) (Issa *et al*., 2005), and different mechanisms can individually or simultaneously exist in colorectal cancer. CIN is the major pathway in CRC pathogenesis, mainly caused by replication stress‐related chromosomal breaks, with structural chromosome abnormalities precipitating chromosome missegregation in mitosis (Burrell *et al*., 2013). It is associated with 65%–75% of sporadic CRC (Pino and Chung, 2010) and featured with an abnormal number of chromosomes (aneuploidy) and loss of heterozygosity (LOH) (Lin *et al*., 2003). Moreover, mutations of a group of specific tumor suppressor genes and oncogenes, such as KRAS, PIK3CA, APC, TP53, and so forth, are found to accumulate in CRCs with the typical karyotypic abnormalities caused by CIN (Colussi *et al*., 2013), though it remains unclear whether these mutations initiate CIN or vice versa.

Nuclear receptor coactivator 3 (NCOA3, also known as SRC3, AIB1, RAC3, or ACTR) is a member of the p160/SRC coactivator family and has intrinsic histone‐acetyltransferase (HAT) activity. It binds nuclear receptors in a hormone‐dependent fashion, remodels chromatin DNA to become more accessible to the transcription machinery, recruits additional transcription factors and coregulators, and thus functions as a central player in the assembly of a coactivator complex to promote gene expression (Anzick *et al*., 1997; Chen *et al*., 1997, 1999; Li *et al*., 1997; Xu *et al*., 2009). NCOA3 has been found to be elevated in breast cancer, liver cancer, prostate cancer, and colorectal cancer, correlated with poor prognosis in most cases, and a vital regulator in the process of tumorigenesis, progression, metastasis, and survival (Anzick *et al*., 1997; Chen *et al*., 2012; Shi *et al*., 2015; Xie *et al*., 2005; Xu *et al*., 2010; Zhou *et al*., 2005). The breast cancer, gastric cancer, and nonsmall cell lung cancer patients with high expression of NCOA3 have significantly shorter overall survival times, indicating that NCOA3 may be an indicator of poor prognosis (Cai *et al*., 2010; Sakakura *et al*., 2000; Zhao *et al*., 2003). However, some studies have reported that amplification of NCOA3 appears to be independently associated with poor prognosis in patients with hepatocellular carcinoma, and moderate or high expression of NCOA3 is associated with poor disease‐specific survival in patients with prostate disease (Gnanapragasam *et al*., 2001; Song *et al*., 2012). Nevertheless, the molecular mechanisms that regulate the tumorigenic functions of NCOA3 in CRC remain unclear so far. To identify protein regulators that interact with NCOA3 in CRC cells, we performed immunoprecipitation coupled with mass spectrometry (IP‐MS) and found mitotic arrest deficient 2‐like protein 2 (MAD2L2, also known as MAD2B or REV7) to be one of the candidates.

Mitotic arrest deficient 2‐like protein 2 is a multifunctional protein with roles in DNA damage repair, cell cycle regulation, gene expression, and carcinogenesis. MAD2L2 was originally identified as a subunit of DNA polymerase ζ critical for DNA translesion synthesis (TLS) (Murakumo *et al*., 2001) and a component of the mitotic spindle assembly checkpoint that inhibits the anaphase‐promoting complex (Chen and Fang, 2001). Recent studies have revealed that MAD2L2 blocks homologous recombination (HR) and promotes nonhomologous end joining (NHEJ) by inhibiting 5’ end resection downstream of 53BP1 and RIF1 and thus functions in DSB repair pathway choices (Boersma *et al*., 2015) (Xu *et al*., 2015). Besides, MAD2L2 has been reported to promote Elk‐1 phosphorylation by c‐Jun N‐terminal protein kinase (JNK) and thus lead to the up‐regulation of Elk‐1 target genes in the presence of DNA damage, which suggests that MAD2L2 might be a central player in coordinating the cellular response to DNA damage (Zhang *et al*., 2007). Moreover, MAD2L2 has been reported to regulate the epigenetic reprogramming of germ cells (Watanabe *et al*., 2013; Zhang *et al*., 2007) and the maintenance of pluripotency in embryonic stem cells (ESCs) (Pirouz *et al*., 2015), and promote the open chromatin configuration through DPPA3 in ESCs (Rahjouei *et al*., 2017). Accordingly, it is not surprising that dysregulation of MAD2L2 has been found in multiple cancers. For instance, MAD2L2 was overexpressed in glioma, epithelial ovarian cancer, and breast cancer (Feng *et al*., 2016; Niimi *et al*., 2014; Zhao *et al*., 2011), while inactivation of MAD2L2 sensitized nasopharyngeal carcinoma cells to DNA‐damaging agents (Cheung *et al*., 2006).

Our study identified MAD2L2 as an interaction protein of NCOA3 and revealed that MAD2L2 suppressed CRC growth both *in vitro* and *in vivo*. Our clinical data indicated that high expression of MAD2L2 was associated with good prognosis in CRC patients. Noteworthily, we showed for the first time that MAD2L2 inhibited CRC development by down‐regulating the protein level of NCOA3. We further demonstrated that increased MAD2L2 expression in CRC cells activated p38, which phosphorylated NCOA3 for its subsequent degradation by the ubiquitin–proteasome pathway. Our study has discovered a novel role of MAD2L2 in regulating the degradation of NCOA3 and suggested that MAD2L2 functions as a tumor suppressor in CRC.

## 2 Materials and methods

### 2.1 Cell culture and chemicals

The human FHC, SW620, SW480, HCT116, HT29, RKO, and DLD1 cells were obtained from American Type Culture Collection (ATCC, Manassas, VA, USA). FHC cells were cultured in DMEM, and the other cells were cultured in RPMI‐1640 supplemented with 10% fetal bovine serum, 100 units·mL^−1^ penicillin, and 100 μg·mL^−1^ streptomycin. All cells were maintained in an incubator with a humidified atmosphere of 95% air and 5% CO_2_ at 37 °C.

MG132, SB203580, cisplatin, and cycloheximide (CHX) were purchased from Sigma (St. Louis, MO, USA) and dissolved in DMSO. The stock solution of MG132 and SB203580 was 10 mm, and the stock solution of CHX was 10 mg·mL^−1^. Methyl methanesulfonate (MMS) was dissolved in PBS, and the concentration of the stock solution was 100 mm. All the stock solutions were stored at −20 °C before use.

### 2.2 siRNA and stable cell lines

The MAD2L2 siRNA and flag‐tagged MAD2L2 overexpression adenovirus were purchased from GenePharma Co., Ltd (Suzhou, China). MAD2L2 knockdown adenovirus was purchased from Hanbio Biotechnology Co., Ltd (Shanghai, China). NCOA3 overexpression adenovirus was purchased from GeneChem (Shanghai, China). Cells were transfected with siRNA duplexes (100 nm) using EndoFectinTM MAX (GeneCopoeia, Inc., Rockville, MD, USA). HCT116 and SW480 cells were used to establish stable cell lines by selection with 1 μg·mL^−1^ puromycin for 4 weeks.

### 2.3 Cell proliferation

Cell viability was determined by MTS assay (Promega Biotech Co., Ltd., Madison, WI, USA). Cells were seeded in 96‐well plates (10 000 cells/well) 24 h after MAD2L2 siRNA transfection. Cell viability was detected 48 h after transfection. Cell viability of stable cell lines with MAD2L2 overexpression was detected 48 h after plating in 96‐well plates (5000 cells/well).

### 2.4 Scratch assay

Cells were transfected with MAD2L2 siRNA, seeded in 6‐well plates, and cultured overnight to a density of 70%–80%. Cell monolayers were scratched with a 100 μL pipette tip and washed with PBS two times to remove detached cells. The scratches were imaged using an Olympus microscope at 0 h, 36 h, and 48 h, respectively, according to their growth rate. The widths of the gap at 0 h (w1) and 36 h or 48 h (w2) were measured, and the relative migration rate was calculated as (w1‐w2)/w1 * 100%.

### 2.5 Tumor‐induced clonogenicity assay

Different stable cell lines were seeded in 6‐well plates (500 cells/well) and incubated. Two weeks later, cells were fixed with formalin and stained by crystal violet. The images of the clones were captured, and the numbers of the clones were counted by the software Image‐Pro Plus 6.0.

### 2.6 RNA extraction and quantitative RT‐PCR (qRT‐PCR)

Total RNA from cells was extracted using RaPure Total RNA Micro Kit (Magen, Guangzhou, China). Endogenous cDNA was generated using ReverTra Ace® qPCR RT Master Mix kit (ToYoBo, Shanghai, China). The primers for qRT‐PCR were purchased from GeneCopoeia, Inc. (Rockville, MD, USA): MAD2L2 (HQP000552), NCOA3 (HQP020041), and GAPDH (HQP006940). qRT‐PCR was performed with the SYBR® Green Real‐time PCR Master Mix (ToYoBo, Shanghai, China).

### 2.7 Antibodies and western blot analysis

Equal amounts of protein lysates were separated by SDS/PAGE and transferred onto polyvinylidene difluoride (PVDF) membranes. The membranes were sequentially incubated with primary and secondary antibodies, and the protein bands were detected by enhanced chemiluminescence. Anti‐MAD2L2, anti‐GAPDH, and anti‐Flag were purchased from Proteintech (Wuhan, China); anti‐NCOA3, anti‐p‐p38, anti‐p‐ERK1/2, anti‐p‐JNK, anti‐ubiquitin, and anti‐histone H3 were from Cell Signaling Technology (Danvers, MA, USA); and antiphosphoserine/threonine was from Bioss (Woburn, MA, USA).

### 2.8 Co‐immunoprecipitation (co‐IP) assays

Protein extracts were prepared and incubated with the antibodies for NCOA3, flag, or IgG for 24 h at 4 °C on a rotating wheel. Then, the sepharose‐conjugated protein‐A/G beads (Santa Cruz Biotechnology, Dallas, TX, USA) were added and incubated at 4 °C for another 24 h on a rotating wheel. After extensive washings with cold PBS containing PMSF, the beads were boiled, and the precipitated proteins were separated by SDS/PAGE and transferred to PVDF membranes for further analysis.

### 2.9 Silver staining and mass spectroscopy (MS)

After electrophoresis, the protein gel was immersed in stationary liquid with 10% acetic acid, 50% ethanol, and 40% water at room temperature on shaker overnight, and then, the protein bands were visualized by the Fast Silver Stain Kit (Beyotime, Haimen, China) and analyzed by MS by Honortech (Beijing, China).

### 2.10 Animal experiments

Female BALB/c nude mice (4 weeks old) were purchased from Vital River Laboratory Animal Technology Co., Ltd. (Beijing, China) and quarantined for 1 week before use for tumor formation experiments. All animal experiment procedures were approved by the Animal Care and Use Committee of Sun Yat‐sen University, and every effort was made to reduce the suffering of animals. 3 × 10^6^ cells were suspended in 100 μL of PBS and subcutaneously injected into BALB/c mice. The weight of the mice and the volume of the tumors were measured every 2 days for 3 weeks. At the end of the experiments, the mice were sacrificed, and the tumors were excised, photographed, and processed for immunohistochemical analyses.

### 2.11 Immunohistochemistry (IHC)

Tissue microarrays with 190 samples were purchased from Outdo Biotech Co., Ltd (Shanghai, China). The microarrays were incubated with anti‐MAD2L2 and anti‐NCOA3 primary antibodies and secondary antibodies, and after color development, scoring was done. The antibodies’ specificity against MAD2L2 and NCOA3 was detected in Fig. S1.

### 2.12 Statistical analysis

Statistical analyses were performed using the SPSS statistical software package (version 17.0). Chi‐square test and *t*‐test were applied for variance analysis, Spearman rank correlation method was for correlation analysis, and Kaplan–Meier analysis was for survival analysis. The mean ± SEM was calculated by GraphPad Prism 6.0 and presented in graphs. *P* < 0.05 was considered statistically significant.

## 3 Results

### 3.1 MAD2L2 was inversely correlated with NCOA3 and predicted favorable prognosis in colorectal cancer (CRC) patients

**Figure 1:**
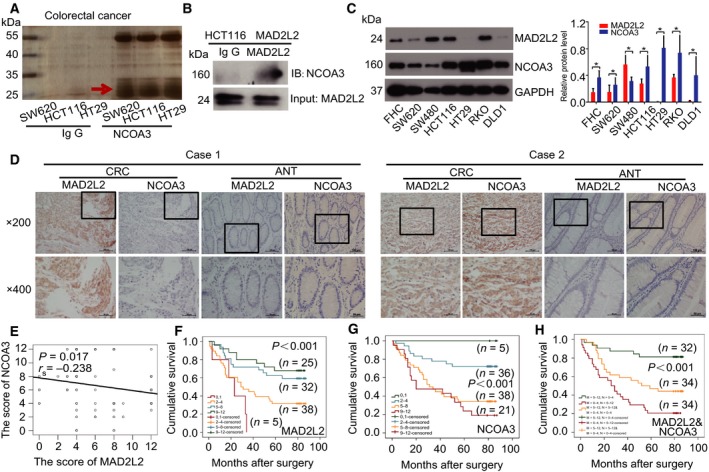
MAD2L2 expression was inversely correlated with NCOA3 expression and predicted favorable prognosis in CRC patients. (A) NCOA3 interaction partners were identified in SW620, HCT116, and HT29 cells stably overexpressing NCOA3 by IP‐MS. (B) The interaction between MAD2L2 and NCOA3 was confirmed by co‐IP in HCT116 cells with stable MAD2L2 overexpression. (C) The basic expression of MAD2L2 and NCOA3 in FHC colon epithelial cells and different CRC cells (SW620, SW480, HCT116, HT29, RKO, and DLD1) and the relative protein level in different CRC cells were shown in C right. (D) Representative images of MAD2L2 and NCOA3 expression in CRC tissues and adjacent normal tissues (ANT). (E) The expression of MAD2L2 and NCOA3 was negatively correlated with CRC tissues. (F) High expression of MAD2L2 was associated with good prognosis in CRC patients. (G) High expression of NCOA3 was related to poor prognosis in CRC patients. (H) High expression of MAD2L2 with low expression of NCOA3 (green curve) predicted favorable prognosis in CRC patients, compared with low expression of MAD2L2 and high expression of NCOA3 (red curve), along with both low and high expression of MAD2L2 and NCOA3 (yellow curve). M means MAD2L2 and N means NCOA3.

To identify protein regulators that interacted with NCOA3 in CRC cells, we performed immunoprecipitation combined with mass spectrometry (IP‐MS) in SW620, HCT116, and HT29 CRC cells with IgG control or NCOA3 antibodies. MAD2L2, with two signal peptide sequences of LIPLK and EVYPVGIFQK, was found to be a candidate interacting with NCOA3 (Fig. 1A), and the interaction between NCOA3 and MAD2L2 was further confirmed by co‐IP in HCT116 cells (Fig. 1B). Next, the expression of MAD2L2 and NCOA3 in a panel of CRC cell lines were detected by western blot, and the basic expression levels of MAD2L2 and NCOA3 in CRC cells tend to be inversely correlated (Fig. 1C).

We then examined whether the expression of MAD2L2 and NCOA3 was also correlated with CRC patients. Immunohistochemical (IHC) analysis revealed that both proteins had significantly higher expression in CRC tissues than in adjacent normal tissues (ANT) (*P *<* *0.001, Table 1), and the patients with high MAD2L2 expression tend to have a low level of NCOA3 (Fig. 1D case 1), while the patients with low MAD2L2 expression tend to have a high level of NCOA3 (Fig. 1D case 2), and the Spearman rank correlation analysis showed that MAD2L2 was in inverse correlation with NCOA3 (*r*_s_ = −0.238, *P *=* *0.017) (Fig. 1E). Moreover, the Kaplan–Meier survival analysis of 100 CRC patients showed that high levels of MAD2L2 predicted favorable prognosis (Fig. 1F), high expression of NCOA3 was associated with poor prognosis (Fig. 1G), and patients with high MAD2L2 combined with low NCOA3 had the best outcome, whereas patients with low MAD2L2 combined with high NCOA3 had the worst outcome (Fig. 1H).

**Table 1:** The expression of MAD2L2 and NCOA3 in CRC is higher than ANT

Variable	CRC	ANT	*χ* ^2^	*P*
	*n* (%)	*n* (%)		
MAD2L2				
−/1+	43 (43)	58 (72.5)	15.71	<0.001
2 + /3+	57 (57)	22 (27.5)		
NCOA3				
−/1+	41 (41)	69 (86.3)	38.29	<0.001
2 + /3+	59 (59)	11 (13.7)		

We further investigated the relationship between the expression of MAD2L2 and NCOA3 and the patients’ clinicopathological characteristics. As is shown in Table 2, high MAD2L2 expression was correlated with small tumor volume (*P *=* *0.017), superficial infiltration (*P *=* *0.023), rare metastasis (*P *=* *0.008), and good clinical staging (*P *=* *0.046). In contrast, high expression of NCOA3 was correlated with deep infiltration (*P *=* *0.026).

**Table 2:** Correlation between MAD2L2, NCOA3, and clinicopathological characteristics with CRC

Variable	*n*	MAD2L2		*χ* ^2^	*P*	NCOA3		*χ* ^2^	*P*
		−/1+	2 + /3+			−/1+	2 + /3+		
Age									
< 70	55	27 (49.1)	28 (50.9)	1.85	0.174	27 (49.1)	28 (50.9)	3.308	0.069
≥ 70	45	16 (35.6)	29 (64.4)			14 (31.1)	31 (68.9)		
Gender									
Male	58	27 (46.6)	31 (53.4)	0.711	0.399	22 (37.9)	36 (62.1)	0.538	0.463
Female	42	16 (38.1)	26 (61.9)			19 (45.2)	23 (54.8)		
Pathological type									
Canalicular adenoma	83	34 (41.0)	49 (59.0)	0.826	0.363	32 (38.6)	51 (61.4)	1.207	0.272
Mucinous adenocarcinoma	17	9 (52.9)	8 (47.1)			9 (52.9)	8 (47.1)		
Pathological grade									
I+II	70	32 (45.7)	38 (54.3)	0.701	0.402	28 (40.0)	42 (60.0)	0.096	0.756
III	20	1 (36.7)	19 (63.3)			13 (43.3)	17 (56.7)		
Tumor volume (cm^3^)									
< 30	51	16 (31.4)	35 (68.6)	5.741	0.017	25 (49.0)	26 (51.0)	2.767	0.096
≥ 30	49	27 (55.1)	22 (44.9)			16 (32.7)	33 (67.3)		
General type									
Infiltrate type	25	10 (40.0)	15 (60.0)	1.851	0.604	10 (40.0)	15 (60.0)	1.542	0.673
Gel type	8	4 (50.0)	4 (50.0)			4 (50.0)	4 (50.0)		
Ulcerative type	47	18 (38.3)	29 (61.7)			21 (44.7)	26 (55.3)		
Protrude type	20	11 (55.0)	9 (45.0)			6 (30.0)	14 (70.0)		
Tumor location									
Left hemicolon	47	20 (42.6)	27 (57.4)	0.007	0.932	15 (31.9)	32 (68.1)	3.026	0.082
Right hemicolon	53	23 (43.4)	30 (56.6)			26 (49.1)	27 (50.9)		
Depth of invasion									
T1/T2/T3	68	24 (35.3)	44 (64.7)	5.148	0.023	33 (48.5)	35 (51.5)	4.98	0.026
T4	32	19 (59.4)	13 (40.6)			8 (25.0)	24 (75.0)		
Lymph node metastases									
N0	52	23 (44.2)	29 (55.8)	0.067	0.796	20 (38.5)	32 (61.5)	0.289	0.591
N1/N2/N3	48	20 (41.7)	28 (58.3)			21 (43.7)	27 (56.3)		
Distant metastasis									
M0	95	38 (40.0)	57 (60.0)	6.977	0.008	39 (41.1)	56 (58.9)	0.002	0.963
M1	5	5 (100)	0 (0)			2 (40.0)	3 (60.0)		
Clinical staging									
I	4	1 (25.0)	3 (75.0)	8.001	0.046	1 (25.0)	3 (75.0)	0.518	0.915
II	47	21 (44.7)	26 (55.3)			19 (40.4)	28 (59.6)		
III	44	16 (36.4)	28 (63.6)			19 (43.2)	25 (56.8)		
IV	5	5 (100)	0 (0)			2 (40.0)	3 (60.0)		

Altogether, these results indicated that the expression of MAD2L2 was inversely related to that of NCOA3 in CRC, and MAD2L2 was associated with good prognosis in CRC patients, which suggested that MAD2L2 might inhibit the development of CRC.

### 3.2 MAD2L2 inhibited the proliferation, clonogenicity, and migration of CRC cells by down‐regulating NCOA3

**Figure 2:**
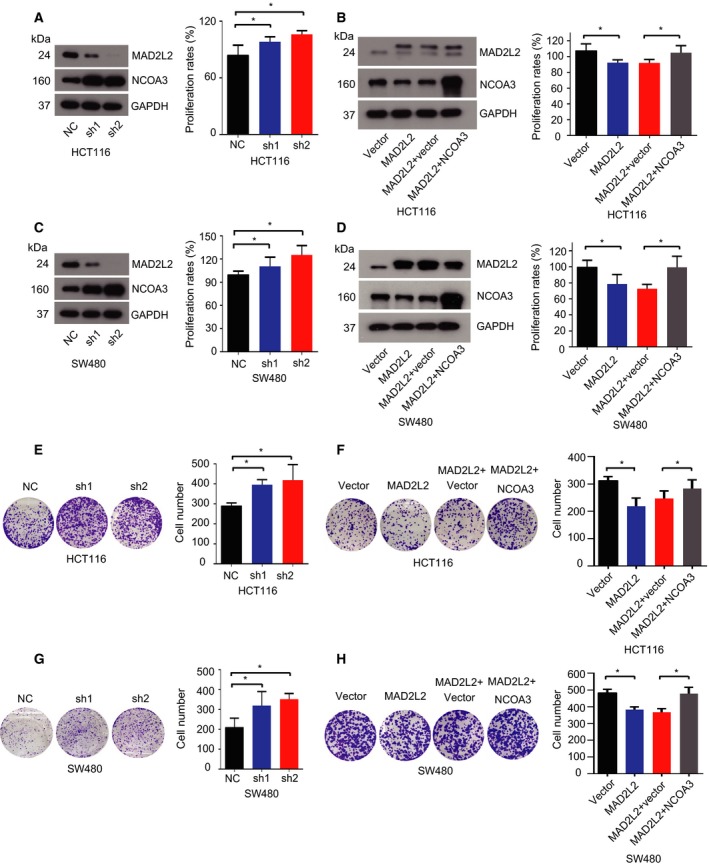
MAD2L2 suppressed CRC cell proliferation and inhibited the clonogenicity of CRC cells by down‐regulating NCOA3. (A,C) Knockdown of MAD2L2 elevated the expression of NCOA3 and promoted the proliferation of HCT116 and SW480 cells * *P *<* *0.05 (*n* = 3). (B,D) Overexpression of MAD2L2 inhibited the expression of NCOA3 and suppressed CRC cell proliferation, which was reversed by NCOA3 overexpression. * *P *<* *0.05 (*n* = 3). (E, G) Knockdown of MAD2L2 increased the clonogenicity of CRC cells. * *P *<* *0.05 (*n* = 3). (F, H) Overexpression of MAD2L2 inhibited the clonogenicity of CRC cells, which was reversed by NCOA3 overexpression. * *P *<* *0.05 (*n* = 3).

**Figure 3:**
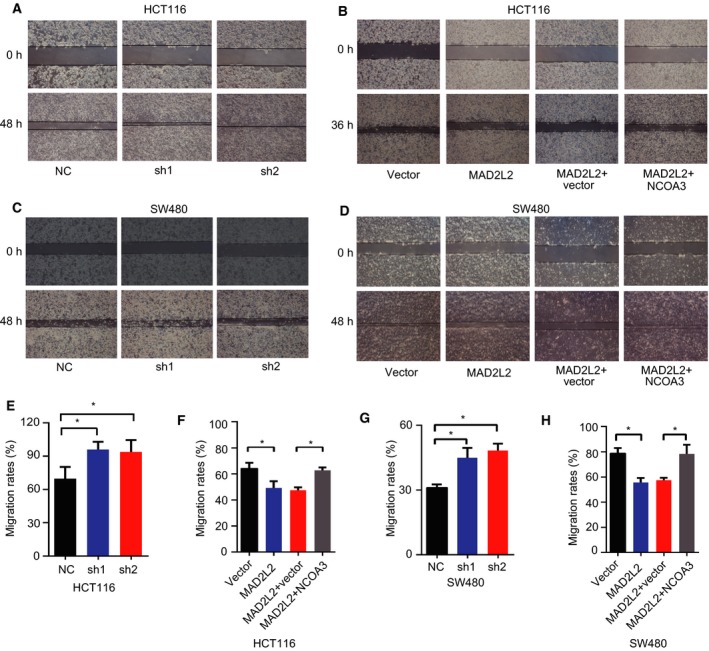
MAD2L2 inhibited CRC cell migration. (A,C,E,G) Knockdown of MAD2L2 promoted CRC cell migration. (B,D,F,H) Overexpression of MAD2L2 inhibited CRC cell migration, which was reversed by NCOA3 overexpression. * *P *<* *0.05 (*n* = 3).

**Figure 4:**
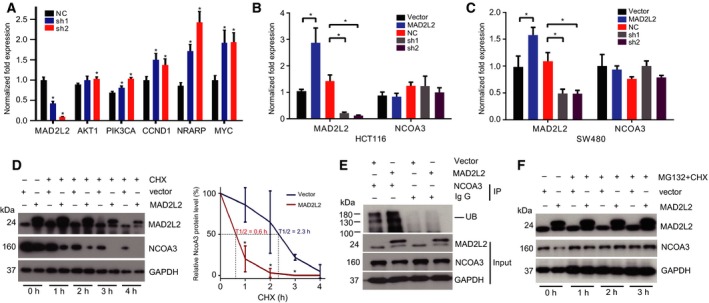
MAD2L2 promoted the degradation of NCOA3 in CRC cells. (A) The RNA levels of known downstream target genes of NCOA3 when MAD2L2 was knocked down in HCT116 cells. * *P *<* *0.05 (*n* = 3). (B, C) NCOA3 mRNA remained unchanged when MAD2L2 was overexpressed (MAD2L2) or knocked down (sh1, sh2) in HCT116 and SW480 cells. * *P *<* *0.05 (*n* = 3). (D) HCT116 cells without (vector) or with MAD2L2 overexpression (MAD2L2) were treated with 1 μg·mL^−1^ CHX for 0, 1, 2, 3, and 4 h, and the expression of NCOA3 was detected by western blot. The MAD2L2‐induced degradation of NCOA3 was enhanced in MAD2L2 overexpression cells. The half‐life curve of NCOA3 showed a considerable short half‐life of 0.6 h, compared to the vector cells with a half‐life of 2.3 h. * *P *<* *0.05 (*n* = 3). (E) Extracts from HCT116 cells without or with MAD2L2 overexpression were immunoprecipitated with NCOA3 antibodies and analyzed for NCOA3 ubiquitination by western blot. (F) The MAD2L2‐induced degradation of NCOA3 was inhibited by MG132 (0.5 μm). CHX (2 μm) was used to inhibit protein synthesis.

To investigate the effect of MAD2L2 on CRC development, we knocked down MAD2L2 with its specific shRNA in HCT116 and SW480 cells and found that MAD2L2 knockdown promoted the proliferation of CRC cells and western blots showed that the level of NCOA3 was elevated when MAD2L2 was down‐regulated (Fig. 2A,C). In contrast, overexpression of MAD2L2 inhibited CRC cell proliferation, which was reversed by overexpression of NCOA3, whereas NCOA3 expression was repressed when MAD2L2 was overexpressed (Fig. 2B,D). Next, clonogenicity experiment showed that knockdown of MAD2L2 increased the clonogenicity of CRC cells (Fig. 2E,G), while overexpression of MAD2L2 decreased the cell clonogenicity and this decrease was reversed by NOCA3 overexpression (Fig. 2F,H). Finally, scratch assay showed that knockdown of MAD2L2 accelerated the migration rate of CRC cells (Fig. 3A,C,E, and G), while overexpression of MAD2L2 reduced the cell migration rate and this reduction was reversed by NCOA3 overexpression (Fig. 3B,D,F and H). To explore the downstream target genes of NCOA3 on CRC development, we knocked down MAD2L2 in HCT116 cells and found that NCOA3 activates the key targets of PI3K/AKT and Notch signaling pathway in RNA levels, which are involved in CRC progression (Fig. 4A). Collectively, these cellular experiments supported the inhibitory role of MAD2L2 in CRC development and suggested that MAD2L2 functioned as a tumor suppressor in CRC through the down‐regulation of NCOA3.

### 3.3 MAD2L2 promoted NCOA3 degradation in CRC cells

To explore the molecular mechanism by which MAD2L2 down‐regulated the expression of NCOA3, we first tested whether MAD2L2 suppressed the transcription of NCOA3. As is shown in Fig. 4B,C, the mRNA level of NCOA3 remained largely unchanged when MAD2L2 was overexpressed or knocked down, which indicated that MAD2L2 did not regulate NCOA3 on the transcription level. We then tested whether MAD2L2 regulated the protein turnover of NCOA3. HCT116 cells without (vector) or with MAD2L2 overexpression (MAD2L2) were treated with 1 μg·mL^−1^ CHX for 0, 1, 2, 3, and 4 h to inhibit protein synthesis, and the expression of NCOA3 was detected by western blot. Excitingly, we found that NCOA3 was stilled detected in the control cells 4 h after CHX treatment (Fig. 4D), but it became barely detectable in cells overexpressing MAD2L2 only 2 h after CHX treatment (Fig. 4D). The half‐life curve of NCOA3 showed a considerable short half‐life of 0.6 h, compared to the vector cells with a half‐life of 2.3 h. These results indicated that overexpression of MAD2L2 accelerated the degradation of NCOA3.

Next, we examined whether MAD2L2 induced NCOA3 degradation by the ubiquitin–proteasome pathway. Immunoprecipitation (IP) experiments were performed with NCOA3 antibodies in HCT116 cells, and the amount of ubiquitinated NCOA3 was larger in cells overexpressing MAD2L2 (Fig. 4E). Moreover, the degradation of NCOA3 induced by MAD2L2 overexpression was inhibited by the proteasome inhibitor MG132 (Qiang *et al*., 2017) (Fig. 4F), confirming that NCOA3 was degraded by the proteasome.

### 3.4 MAD2L2 activated p38 to phosphorylate NCOA3 for its subsequent ubiquitination and degradation

**Figure 5:**
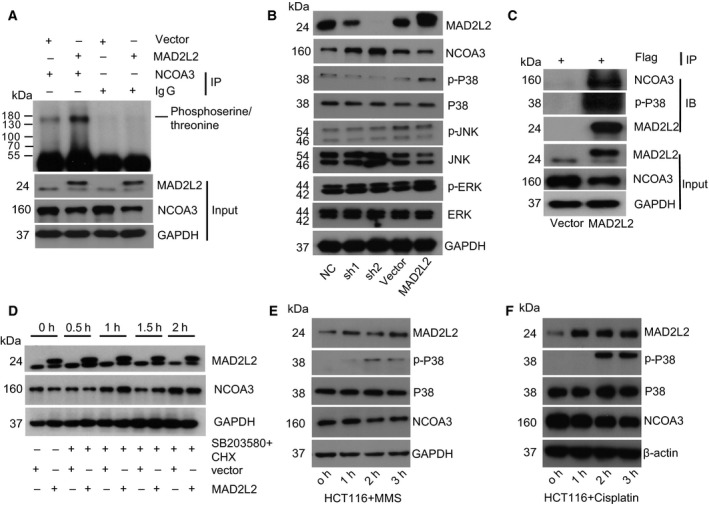
MAD2L2 activated p38 to phosphorylate NCOA3 for its subsequent ubiquitination and degradation. (A) Extracts from HCT116 cells without or with MAD2L2 overexpression were immunoprecipitated with NCOA3 antibodies and analyzed for NCOA3 phosphorylation by western blot. (B) The levels of p‐p38, p‐JNK, and p‐ERK in HCT116 cells with MAD2L2 knockdown or overexpression were detected by western blot. (C) The interaction among MAD2L2, NCOA3, and p‐p38 was detected by co‐IP in HCT116 cells overexpressing flag‐tagged MAD2L2. (D) The MAD2L2‐induced degradation of NCOA3 was inhibited by p‐p38 inhibitor SB203580 (0.5 μm). CHX (2 μm) was used to inhibit protein synthesis. (E) The DNA‐damaging agent MMS induced MAD2L2 expression and subsequent p38 activation and NCOA3 degradation. (F) The DNA‐damaging chemotherapy drug cisplatin (4 μg·mL^−1^) induced MAD2L2 expression and subsequent p38 activation and NCOA3 degradation.

Previous studies had reported that ubiquitination of NCOA3 was mediated by its phosphorylation (Ferry *et al*., 2011; Gianni *et al*., 2006; Wu *et al*., 2007), so we investigated whether MAD2L2 affected the phosphorylation of NCOA3. IP with NCOA3 antibodies in HCT116 cells showed that the phosphorylation of NCOA3 was enhanced in cells with MAD2L2 overexpression (Fig. 5A). Multiple kinases can differentially phosphorylate NCOA3, and it has several MAPK phosphorylation sites (Wu *et al*., 2004). We therefore tested whether MAD2L2 activated p38, JNK, or ERK1/2 to phosphorylate NCOA3. As is shown in Fig. 5B, the level of p‐p38 was positively correlated with the expression MAD2L2 and negatively with NCOA3. Furthermore, we detected interaction among MAD2L2, NCOA3, and p‐p38 by co‐IP in HCT116 cells with flag‐tagged MAD2L2 overexpression (Fig. 5C). In addition, the p38 kinase inhibitor SB203580 consistently inhibited the degradation of NCOA3 induced by MAD2L2 (Fig. 5D). Collectively, these findings supported our hypothesis that MAD2L2 activated p38 to phosphorylate NCOA3, which primed NCOA3 for the subsequent ubiquitination and degradation by the proteasome.

Considering that chromosome instability is the major cause of CRC (Pino and Chung, 2010), and the expression of MAD2L2 was higher in CRC cells and tissues (Fig. 1C, Table 1), we presumed that MAD2L2 had elevated expression in response to DNA damages to serve as a protective factor in CRC. Cisplatin, a DNA‐damaging chemotherapy drug in the clinical treatment of colorectal cancer, can interact with the DNA guanine bases and prevent the replication of DNA. We treated HCT116 cells with the DNA‐damaging agent MMS and cisplatin and found that the expression of MAD2L2 was significantly increased within 1 h after MMS treatment, while noticeable p38 activation and NCOA3 degradation were detected about 2 h after MMS and cisplatin treatment (Fig. 5E,F).

### 3.5 MAD2L2 knockdown promoted CRC growth in a mouse xenograft model

**Figure 6:**
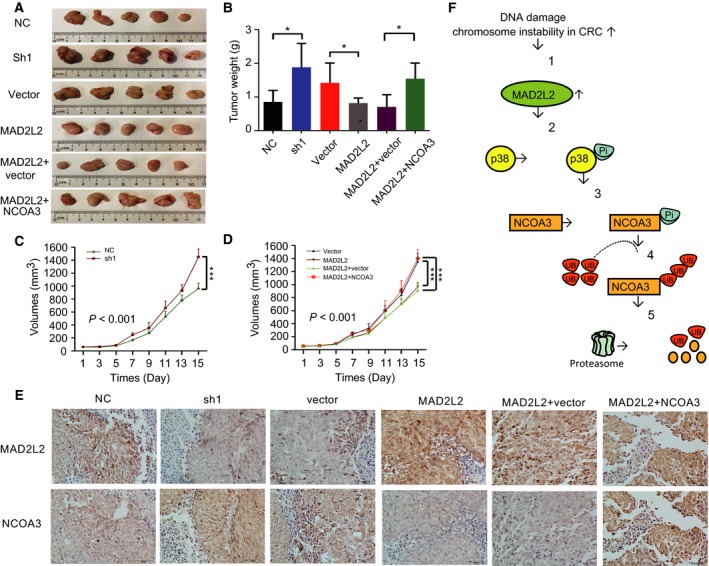
MAD2L2 knockdown promoted CRC growth in a mouse xenograft model and MAD2L2‐regulated NCOA3 phosphorylation, ubiquitination, and degradation in CRC cells. Nude mice were subcutaneously injected with HCT116 cells with nonspecific siRNA (NC), MAD2L2 knocked down by its specific shRNA (sh1), vector, MAD2L2 overexpression (MAD2L2), MAD2L2 +  vector, MAD2L2 +  NCOA3 overexpression (NCOA3). (A) Images of the CRC tumor xenograft from each mouse (*n* = 5 mice/group). (B) Tumor weights were analyzed. *P <* *0.05 (*n* = 5) (C, D) Tumor volumes were recorded and analyzed. ***P <* *0.001 (*n* = 5) (E) The expression of MAD2L2 and NCOA3 in tumor tissues was analyzed by IHC staining. (F) (1) MAD2L2 had elevated expression in response to the increased DNA damage and chromosome instability in CRC cells. (2) MAD2L2 activated p38. (3) p‐p38 phosphorylated NCOA3. (4) Phosphorylated NCOA3 was ubiquitinated. (5) Ubiquitinated NCOA3 was degraded by the proteasome.

The significant association of MAD2L2 with NCOA3 expression revealed in cellular experiments and clinical outcomes led us to further verify the roles of MAD2L2 and NCOA3 in CRC in a mouse xenograft model. HCT116 cells were subcutaneously injected into the left flank of nude mice, and tumors developed at the injection sites after 1 week. Tumor volumes were measured and recorded every 2 days, and the tumor xenografts were harvested, weighed, and processed for IHC staining 3 weeks after CRC cell injection. As is shown in Fig. 6A‐D, MAD2L2 knockdown promoted tumor growth, while MAD2L2 overexpression inhibited tumor growth, and this inhibition was rescued by elevated NCOA3 expression. IHC analysis showed that the levels of MAD2L2 and NCOA3 were inversely correlated (Fig. 6E). These *in vivo* results were consistent with our *in vitro* observations and confirmed the tumor suppressor role of MAD2L2 in CRC.

## 4 Discussion

Accumulating evidence shows that NCOA3 is highly expressed in a various human cancers (Anzick *et al*., 1997; Xu *et al*., 2010; Zhou *et al*., 2005), and it can interact with nuclear receptors and other transcription factors to regulate the expression of their target genes involved in many signaling pathways, including EGFR, Akt, MAPK, E2F1, and Notch (Long *et al*., 2012; Louie *et al*., 2004; Mo *et al*., 2015; Yan *et al*., 2006). However, little is known about how NCOA3 is regulated in colorectal cancer (CRC). In this study, we discovered that MAD2L2 interacted with NCOA3 and regulated its protein level in CRC. MAD2L2 is a regulatory subunit of DNA polymerase ζ that is involved in DNA translesion synthesis (TLS) (Boersma *et al*., 2015), and the alterations of MAD2L2 are implicated in the pathogenesis of a wide variety of tumors (Feng *et al*., 2016; Niimi *et al*., 2014; Okina *et al*., 2015). Our *in vivo* and *in vitro* results showed that MAD2L2 suppressed CRC development by down‐regulating NCOA3, and our clinical data suggested that MAD2L2 predicted favorable prognosis in CRC patients. Our mechanism study showed that MAD2L2 had increased expression in the presence of DNA damage and activated p38 to phosphorylate NCOA3 for its subsequent degradation by the ubiquitin–proteasome pathway.

Colorectal cancer is one of the most common cancers and continued to be a serious public health problem in clinic. To provide valuable information for the clinical outcome prediction, we analyzed the expression of MAD2L2 and NCOA3 in CRC patients. Our results showed that there was a reverse correlation between MAD2L2 and NCOA3 expression in CRC tissues (Fig. 1D,E), which was in accordance with our findings in CRC cells (Fig. 1C). Moreover, higher expression of MAD2L2 was associated with lower tumor volume, earlier TNM stage, less invasion, and a smaller chance of distant metastasis in CRC patients (Table 2), which suggested that MAD2L2 was a suppressor of CRC growth and metastasis. Consistently, survival analysis indicated that MAD2L2 suppressed but NCOA3 promoted CRC development (Fig. 1F,G). Interestingly, the expression of both MAD2L2 and NCOA3 was higher in CRC tissues than normal tissues (Table 1). Given that CRC cells have increased DNA damage and chromosome instability (Guo *et al*., 2016; Ribeiro *et al*., 2008; Xia *et al*., 2016), and MAD2L2 plays a critical role in DNA repair, we proposed that the expression of MAD2L2 was elevated in CRC tissues as a stress response, and this was supported by our result that MMS and cisplatin treatment induced MAD2L2 expression (Fig. 5E,F). Collectively, our data have revealed that MAD2L2 is a protective factor in the pathogenesis of CRC.

To further study the biological relationship between MAD2 l2 and NCOA3, we knocked down or overexpressed MAD2L2 in CRC cells to determine the effects of MAD2L2 on the protein level of NCOA3, cell proliferation, colony formation, and migration capacity. Our data demonstrated that knockdown of MAD2L2 increased NCOA3 expression and enhanced the proliferation, colony formation, and migration of CRC cells, whereas overexpression of MAD2L2 had the opposite effects, which were reversed by NCOA3 overexpression (Fig. 2,3 and 6A‐D). Consistent with our findings, knockdown of NCOA3 decreased cell proliferation, colony formation, and tumorigenesis of CRC cells *in vitro* and *in vivo* (Mo *et al*., 2015), suggested that MAD2L2 was a novel regulator of NCOA3 in CRC progression. However, the effects of MAD2L2 on cell proliferation were not the only suppressor mechanism, and it has been reported that other mechanisms also play an important role in tumorigenesis of CRC cells (Kramer *et al*., 2016; Siraj *et al*., 2017).

To validate that the observed effects on proliferation and migration are reflected at the functional level of NCOA3, the mRNA levels of known downstream target genes of NCOA3 were detected when MAD2L2 was knocked down in HCT116 cells (Fig. 4C). Studies have shown NCOA3 activates the PI3K/AKT pathway and its downstream effectors in mammary tumor cells derived from AIB1‐tg mice (Torres‐Arzayus *et al*., 2004). As the key genes of PI3K/AKT pathway, the mRNA levels of AKT1, PIK3CA, and CCND1 were significantly increased, suggested that NCOA3 promotes CRC progression through regulating the PI3K/AKT pathway‐related genes. Increasing evidence has shown that Notch signaling is related to CRC progression, and NRARP represents Notch signaling activity in CRC (Kim *et al*., 2012; Mo *et al*., 2015). Moreover, Notch signaling can directly activate MYC, and a protooncogene holds a central role in regulating tumor growth (Jitschin *et al*., 2015; Xiao *et al*., 2011). Our study found that the mRNA levels of NRARP and MYC was significantly elevated, and revealed that typical target gene of Notch signaling plays an important role in CRC development. Further study showed that MAD2L2 did not regulate NCOA3 on the transcription level (Fig. 4B,C), but promoted the protein degradation of NCOA3 (Fig. 4D). Moreover, we confirmed that the degradation of NCOA3 induced by MAD2L2 happened through the ubiquitin–proteasome pathway (Fig. 4E, 5A), which controls the degradation of the majority of regulatory proteins in mammalian cells (Naujokat and Saric, 2007; Vriend and Reiter, 2015). Previously, phosphorylation of NCOA3 was found to promote its ubiquitination and degradation (Ferry *et al*., 2011; Wu *et al*., 2007). NCOA3 can be phosphorylated by kinases including MAPKs, GSK3, PKA, and CKI (Wu *et al*., 2004). Among them, MAPKs are key signaling molecules in cell growth, proliferation and development, and functionally important for NCOA3 phosphorylation (Ferry *et al*., 2011). Extracellular signal‐regulated kinase (ERK), c‐Jun N‐terminal protein kinase (JNK), and p38 kinase are the three major MAPKs (Chang and Karin, 2001), and Wu *et al*. found that p38 and JNK were able to phosphorylate multiple sites of NCOA3 (Wu *et al*., 2004). In this study, we identified that p38 was the chief mediator of MAD2L2‐induced NCOA3 ubiquitination and degradation (Fig. 5B‐D). Here, we propose a model for the MAD2L2‐regulated NCOA3 phosphorylation, ubiquitination, and degradation in CRC cells (Fig. 6F): In response to the increased DNA damage and chromosome instability in CRC cells, MAD2L2 had elevated expression and activated p38, which then phosphorylated NCOA3 for subsequent degradation through the ubiquitin–proteasome pathway.

In summary, we have discovered that MAD2L2 inhibited CRC development by promoting NCOA3 degradation. Our work has demonstrated that modulation of the MAD2L2 gene product has the potential to become a new therapy for CRC.

## Author contribution

YL, LL, MC, XY, XW, and WD conceived and designed the project; YL, LL, MC, and ZG acquired the data; YL, LL, MC, ZG, HQ, GQ, QL, XF, TL, WL, DS, ML, TK, WH, and XW analyzed and interpreted the data; YL, MC, and WD wrote the paper; WD supervised the project.

## Ethics approval

All animal procedures were performed following the Guide for the Care and Use of Laboratory Animals (NIH publication Nos. 80‐23, revised 1996) and the Institutional Ethical Guidelines for Animal Experiments developed by Sun Yat‐sen University.

## Supporting information


**Fig. S1.** The specificity of the antibodies against MAD2L2 and NCOA3 were examined in CRC cells. MAD2L2 and NCOA3 proteins were detected by their antibodies by western blots of the entire gel with SW620, SW480 and HCT116 cell extracts.
