# Supplementary figures and images for: MAD2L2 inhibits colorectal cancer growth by promoting NCOA3 ubiquitination and degradation
Source: Mol Oncol. 2018 Feb 13;12(3):391–405. doi: 10.1002/1878-0261.12173 (PMC5830628; doi:10.1002/1878-0261.12173)

**Figure S1**

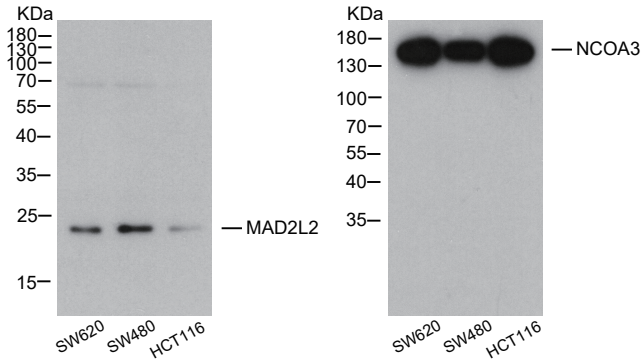

Supplement: Supplementary file 1 — Fig. S1. The specificity of the antibodies against MAD2L2 and NCOA3 were examined in CRC cells. MAD2L2 and NCOA3 proteins were detected by their antibodies by western blots of the entire gel with SW620, SW480 and HCT116 cell extracts. [file MOL2-12-391-s001.pdf]
